# Association between Alcohol Consumption and Body Mass Index in University Students

**DOI:** 10.31372/20190401.1035

**Published:** 2019

**Authors:** Uraiporn Booranasuksakul, Alongkote Singhato, Narisa Rueangsri, Piyapong Prasertsri

**Affiliations:** a Faculty of Allied Health Sciences, Burapha University, Thailand

**Keywords:** alcohol consumption, overweight, obesity, university students

## Abstract

*Objective*: The aim of this study was to determine the correlation between alcohol consumption and body mass index in university students in Eastern Thailand.

*Methods*: Undergraduate students (19–23 years, *n* = 396) were randomly surveyed via questionnaires, which included general information, alcohol consumption, and unhealthy food consumption. Average daily alcohol consumption was then calculated from grams of ethanol consumed per day. A subject, who has body mass index (BMI) more than 23 kg/m^2^, was defined as excessive weight. Difference between genders of each variable was compared using independent *t*-test. Mean of each variable between groups was compared using analysis of variance (ANOVA). The correlation between average daily alcohol consumption and BMI, unhealthy consumption and BMI were analyzed by applying Pearson correlation coefficient.

*Results*: 229 university students consumed alcohol (58%). After 229 subjects were divided into three categories, the average daily alcohol consumption of the overweight group was significantly higher than the underweight and normal weight groups in women; meanwhile, unhealthy food consumption frequencies was not different between groups. Average daily alcohol consumption levels for overweight group were 74.17 and 73.45 g/day in men and women, respectively. Furthermore, higher daily alcohol consumption was independently associated with higher BMI (95% confidence interval [CI] *R* = 0.161: *p* = 0.015; men *R* = 0.120: *p* = 0.236; women *R* = 0.214: *p* = 0.015).

*Conclusion*: There was a positive relationship between alcohol consumption and BMI in university students in Eastern Thailand. This study supports that the daily alcohol consumption is a risk factor for excessive weight and gender may contribute to the correlation.

## Introduction

Overweight and obesity are major health problems across the world (Bray, Kim, Wilding, & World Obesity Federation, 2017). Causes of obesity are an excessive energy intake from food, low physical activity levels (Ridgers, Saint-Maurice, Welk, Siahpush, & Huberty, 2014), and several other environmental factors (Bray et al., 2017). Obesity is a principal contributing factor in the development of metabolic syndrome (MetS) and excess visceral adiposity is a major risk of MetS (Hall, Wang, do Carmo, Kamimura, & Hall, 2018; Rankin, Blood-Siegfried, Vorderstrasse, & Chlebowy, 2015). In Asian populations, visceral adiposity is associated with the development of MetS even in normal weight (Ding et al., 2018).

Excessive alcohol consumption among the world’s young adults is a major global public health concern while the rising rate of those overweight in addition to obesity among the young is a growing concern (Peltzer et al., 2014; Snyder, Milici, Slater, Sun, & Strizhakova, 2006). Overweight and obesity in young adults and adolescents are linked to greater risk for MetS which is associated with incident diabetes (Stokes et al., 2018). Energy consumed as alcohol is an additive from other dietary sources (Yeomans, 2010). Moreover, increased energy intake with alcohol use may promote a positive energy balance and contributing factor to weight gain (Traversy & Chaput, 2015). Low to moderate alcohol consumption is therefore recommended (Zhao, Stockwell, Roemer, Naimi, & Chikritzhs, 2017). A plethora of studies have demonstrated the relationship between alcohol consumption and adverse health effects (Park, Park, & Hwang, 2017; Rehm, 2011) such as being overweight or obese, and also metabolic syndrome (Pengpid & Peltzer, 2017; Wakabayashi, 2011). Public health problems attributable to harmful alcohol consumption have recently been focused on, as has the demand for global information on alcohol consumption and alcohol-attributable/alcohol-related harm. As a consequence, related policy responses have increased significantly (Poznyak et al., 2014).

Recently, a clear association between alcohol consumption and weight gain has not been apparent based on the mixed, conflicting available evidence on the topic. Given that the relationship between excessive alcohol consumption and obesity is a public health concern, a clear understanding of both is therefore necessary (Traversy & Chaput, 2015). University students are representative of young adults or youth who will later become mature adults who may be particularly at risk of MetS (Robertson, 2018). There is a high prevalence of overweight individuals as well as obesity among university students in Thailand (Pengpid & Peltzer, 2015). However, in Thailand there is limited data concerning the connection between alcohol consumption and obesity in young adults. Accordingly, this study aims to examine the association of alcohol consumption and obesity among university students in Eastern Thailand.

## Methods

### Subjects

This study was a cross-sectional study aimed at examining the association between alcohol consumption and obesity among university students. Students were recruited through announcements and randomly surveyed via questionnaires. Anthropometric measurements (weight and height) were obtained to determine BMI. Questionnaires were used to assess alcohol and unhealthy food consumption. Inclusion criteria were: aged between 19 and 23 years. Individuals were excluded if they did not complete the questionnaires, had health problems such as non-communicable diseases, contagious diseases, or digestive system issues which have an effect on metabolic systems.

### Ethical Statement

All subjects signed a consent form prior to enrollment in the study. The consent form and the study protocols in this study were in accordance with the ethical standards of the Human Ethics Committee of Burapha University (approval no. 10/2559), as well as with the 1964 Helsinki declaration and its later amendments.

### Sample Size Calculation

Sample size in this study was calculated using Taro Yamane formula. Population size was set at 38,517 with margin of error set at 0.05, and 95% confidence interval. Thus, the sample size comprised of 396 subjects.

### Questionnaires

The questionnaires consisted of three parts including general information, alcohol consumption frequency, and unhealthy food consumption frequency. General data contained sex, age, weight, height, health status, and alcohol consumption level. The alcohol consumption questions contained the amount (milliliters per time) and the frequency of each type of alcohol consumption from the previous 12 months (times per day). Frequency categories were 5–6 times per week, 3–4 times per week, 1–2 times per week, 2–3 times per month, and 1 time per month. The unhealthy food frequency questionnaire (FFQ) or unhealthy food consumption contained items of high fat food, high sugar food, high fat and high sugar food, snacks, fast food, processed foods, and sugary drinks. Frequency categories were: 5–6 times per week, 3–4 times per week, 1–2 times per week, 2–3 times per month, and 1 time per month.

### Calculation of Alcohol Consumption

Average daily alcohol consumption (grams of ethanol per day) was calculated by the amount of alcohol consumption (milliliters per day) multiplied by the alcohol percentage in each type and the specific strength of the alcohol (0.79). Average daily alcohol consumption was calculated from the average amount of ethanol consumption per day for all types. All subjects were classified into four groups: non-drinkers; light drinkers: <22 g ethanol/day; heavy drinkers: ≥22 and <44 g ethanol/day; and very heavy drinkers: ≥44 g ethanol/day (Wakabayashi, 2011).

### Measurement of BMI

BMI was calculated as body weight divided by the squared value of height (kg/m^2^). Body weight as well as height measurements were taken using a stadiometer (Health-O-Meter ProSeries, USA). Body weight was measured in the standing position while wearing minimal clothing, with height measured during inspiration.

### Statistical Analysis

All data were calculated using Predictive Analytics Software Statistics (PASW) version 22 (SPSS Inc, Chicago, IL, USA). BMI was calculated utilizing weight and height (normal is defined as 18.5–22.9 kg/m^2^ and overweight is defined as greater than 23.0 kg/m^2^). General information, alcohol consumption, and unhealthy food consumption frequency were analyzed via frequency distribution. Difference between genders of each variable was compared using independent *t*-test. Mean of each variable between groups was compared using analysis of variance (ANOVA) followed by Fisher’s Least-Significant Difference (*p* < 0.05). The correlation between average daily alcohol consumption and BMI, unhealthy consumption and BMI were analyzed by applying Pearson correlation coefficient, with *p*-value < 0.05 considered to be statistically significant for correlation. In addition, adjusted odds ratio with 95% confidence intervals (CIs) for the association between drinking and excess weight were analyzed.

## Results

The data gathered from 396 subjects revealed that BMI and unhealthy food consumption were not different between men and women but alcohol consumption was different between genders. Among drinking groups, the results show that BMI was different between non-drinking group and very heavy drinking group in men and it was different between non-drinking group and heavy drinking group in women. Unhealthy food consumption was different between non-drinking group and light drinking group in women. The average daily alcohol consumption among the very heavy group was significantly higher than the other groups (Table 1).

**Table 1:** Subjects Data and Comparison of each Variable among Groups

	Overall	Drinking group			
	(*N* = 396)	Non	Light	Heavy	Very heavy
		(*n* = 167)	(*n* = 167)	(*n* − 24)	(*n* = 38)
Sex (no.)					
Men	144	44	69	12	19
Women	252	123	98	12	19
BMI (kg/m^2^)					
Mean (SD)					
Men	21.49 (3.73)	20.78 (4.12)	21.43 (3.51)	21.76 (4.09)	23.11 (3.13)^a^
Women	21.23 (3.84)	20.74 (3.91)	21.34 (3.73)	23.34 (3.65)^a^	22.49 (3.59)
Alc./day (g/day)					
Mean (SD)					
Men	32.95 (126.75)^∗^	0.00^b^	6.11 (5.84)^b^	29.87 (6.96)^b^	208.65 (299.11)
Women	18.88 (73.62)	0.00^b^	6.15 (5.75)^b^	29.85 (6.74)^b^	199.77 (193.49)
Unhealthy FFQ					
Mean (SD)					
Men	2.47 (0.69)	2.45 (0.64)	2.47 (0.70)	2.40 (0.67)	2.55 (0.81)
Women	2.38 (0.71)	2.30 (0.70)	2.49 (0.74)^a^	2.57 (0.65)	2.24 (0.65)

*N* = 396. BMI: body mass index; Alc./day: average daily alcohol consumption (grams of ethanol per day); unhealthy FFQ: unhealthy food frequency questionnaire (high risk >3.5). Non-drinkers; light drinkers: <22 g ethanol/day; heavy drinkers: ≥22 and <44 g ethanol/day; very heavy drinkers: ≥44 g ethanol/day.

^*^ Significant differences compared with gender; *p* < 0.05.

^a^ Significant differences compared with non-drinking group; *p* < 0.05.

^b^ Significant differences compared with very heavy drinking group; *p* < 0.05.

Table 2 presents results in accordance with 229 university students from 396 students who consumed alcohol (58%). From those 229 students, there were 129 women and 100 men. Those 229 subjects were divided into three categories as follows: underweight (BMI < 18.5 kg/m^2^), normal weight (BMI 18.5–22.9 kg/m^2^), and overweight (BMI > 23 kg/m^2^); 123 students were of normal weight (54%), 64 were overweight (28%), and 42 were underweight (18%). The average daily alcohol consumption of the overweight group was significantly higher than the underweight and normal weight groups in women; meanwhile, unhealthy food consumption frequencies were not different between groups.

**Table 2:** The Information of Underweight, Normal Weight, and Overweight Subjects who Consumed Alcohol

	Underweight	Normal weight	Overweight
	(BMI < 18.5 kg/m^2^)	(BMI 18.5–22.9 kg/m^2^)	(BMI > 23 kg/m^2^)
	(*n* = 42)	(*n* = 123)	(*n* = 64)
Sex (no.)			
?Men	19	49	32
?Women	23	74	32
Alc./day			
Mean (SD)			
?Men	10.56 (14.17)	44.29 (123.80)	74.17 (215.54)
?Women	9.95 (16.00)	29.43 (81.38)	73.45 (152.63)^a,b^
Unhealthy FFQ			
Mean (SD)			
?Men	2.46 (0.57)	2.40 (0.74)	2.61 (0.76)
?Women	2.29 (0.49)	2.52 (0.81)	2.43 (0.63)

*N* = 229. Alc./day: average daily alcohol consumption (grams of ethanol per day); unhealthy FFQ: unhealthy food frequency questionnaire (high risk > 3.5).

^a^ Significant differences compared with those underweight; *p* < 0.05.

^b^ Significant differences compared with those of normal weight; *p* < 0.05.

**Figure 1:**
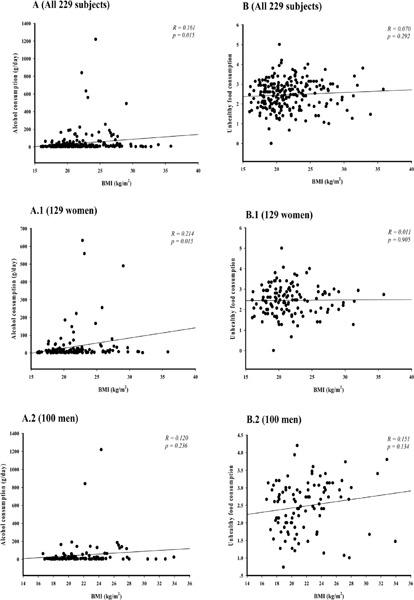

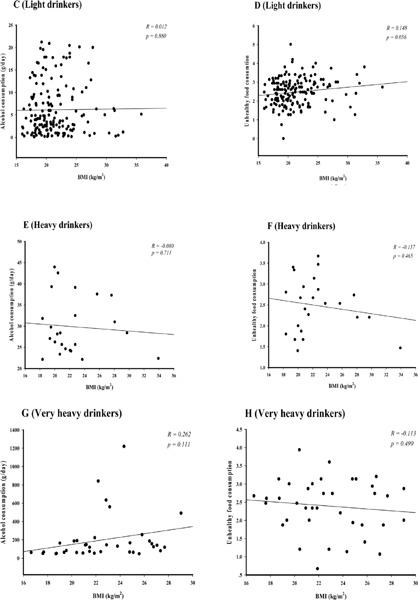
Correlation lines of Pearson’s correlations (*R*) between alcohol consumption and unhealthy food consumption with BMI in subjects who consumed alcohol. *N* = 229; *p* < 0.05, significant differences; light drinkers: <22 g ethanol/day; heavy drinkers: ≥22 and <44 g ethanol/day; very heavy drinkers: ≥44 g ethanol/day.

Figure 1(A)–(H) shows Pearson’s correlations (*R*) between alcohol consumption, unhealthy food consumption, and BMI in subjects who consumed alcohol. There was a small positive correlation between average daily alcohol consumption and BMI (*R* = 0.161, *p* = 0.015) (A). The very heavy drinking group demonstrated greater correlation than the other two groups [light drinking group, *R* = 0.012, *p* = 0.880 (C); heavy drinking group, *R* = -0.080, *p* = 0.711 (E); very heavy drinking group, *R* = 0.262, *p* = 0.111 (G)]. There was a small positive correlation between unhealthy consumption and BMI (*R* = 0.148, *p* = 0.056) (D) in light drinking group. There was a small negative correlation between unhealthy consumption and BMI (*R* = -0.157, *p* = 0.465) (F) in heavy drinking group. The results show that high average daily alcohol consumption was independently associated with an increase in BMI. Moreover, subjects who consumed alcohol had significantly higher prevalence of obesity than those who did not (adjusted odds ratio (OR) 1.64, 95% CI 0.58–4.62).

## Discussion

This study found that average BMI was increased with an increased level of alcohol consumption in men and women. The highest BMI mean (>23 kg/m^2^) were found in heavy women drinkers and very heavy men drinkers which were different from no drinkers with normal BMI. Moreover, the correlation between alcohol consumption and BMI in women was higher than men while the correlation between unhealthy food consumption in men was higher than women. These results show that gender may contribute to the correlation between alcohol consumption and unhealthy food consumption with BMI (Lee, 2012). In some studies, the relationship between moderate alcohol consumption was associated with a lower risk of MetS in both sexes (Djoussé et al., 2004). But in other studies, the relationship was significant only in women (Y.W. Park et al., 2003; Rosell, De Faire, & Hellenius, 2003; Wilsgaard & Jacobsen, 2007; Zhu, St-Onge, Heshka, & Heymsfield, 2004). Furthermore, there were no differences of unhealthy food consumption between BMI groups. These data signify that BMI was related to alcohol consumption. In this study, BMI was highest in the very heavy drinkers group, which conforms to a previous study in Japan which showed significant increases in prevalence of metabolic syndrome in persons consuming alcohol (Wakabayashi, 2011). Several evidential studies also confirmed the relationship between heavy drinking, weight gain, and metabolic syndrome. A cross-sectional study of the Korea National Health and Nutrition Examination Survey 2008 pointed to the notion that drinking higher quantities in addition to frequent binge-drinking were indicators of a higher prevalence of metabolic syndrome (Lee, 2012). A study in a population-based sample of elderly Australian men revealed that a higher alcohol intake was associated with greater total adiposity (Coulson et al., 2013). A study in a Korean population established that there was no relationship between the frequency of alcohol consumption and being overweight or obese in boys and girls. Nevertheless, they discovered that high alcohol consumption could contribute to weight gain in girls (Baek & So, 2012). Besides that, a study in England exhibited a positive association between calories consumed from alcohol and obesity (Shelton & Knott, 2014). Hence, the outcomes of many previous studies support the present study which demonstrates the relationship between alcohol consumption and carrying excess weight.

Even though previous epidemiological studies have shown a positive, negative, or no relationship between alcohol consumption and body weight (Traversy & Chaput, 2015), this study demonstrates a positive relationship between alcohol consumption and being overweight or obese. Our study indicates that daily alcohol consumption is a risk factor for obesity. Alcohol is a source of energy comprising of an energy content of 7.1 kcal/g. Besides that, several metabolic studies showed a suppression of lipid oxidation by alcohol, and therefore the improvement of a positive fat balance with non-oxidized fat deposited differently in the abdominal area (Suter & Tremblay, 2005).

There were several limitations to this study. The classified BMI by Asian criteria (WHO, 2004) for Thai people may be different in other countries. There are differences in body fat distribution and body fat percentages as well as BMI compared to other ethnic groups for metabolic syndrome (Alpert & Thomason, 2016). Hence, the classifications of BMI and obesity need to be distinguished by ethnic groups. This topic still needs to be supported by further studies from other countries to confirm the correlation between alcohol consumption and obesity in young adults. Although gender and unhealthy food consumption were adjusted in multivariate analysis, there are other factors, for instance, physical activity, socioeconomic status, and race/ethnicity, which possibly confound the relationship between BMI and alcohol consumption. Further research ought to employ a larger sample size with regards to the prevalence of daily alcohol consumption amount, as well as the further objective should study more variables of having MetS in the young adults.

## Conclusion

The relationship between excessive alcohol consumption and obesity is a public health concern, but the association between alcohol consumption and body weight have not been clear in many studies. The findings of this study support a relationship between increased daily alcohol consumption and excessive weight in university students in Eastern Thailand and gender may contribute to the correlation.

## Acknowledgment

This research was supported by the Faculty of Allied Health Sciences, Burapha University, Thailand.

## Declaration of Conflicting Interests

No conflict of interest was reported by the authors of this paper.
